# Efficacy of (+)-Lariciresinol to Control Bacterial Growth of *Staphylococcus aureus* and *Escherichia coli* O157:H7

**DOI:** 10.3389/fmicb.2017.00804

**Published:** 2017-05-03

**Authors:** Vivek K. Bajpai, Shruti Shukla, Woon K. Paek, Jeongheui Lim, Pradeep Kumar, Pankaj Kumar, MinKyun Na

**Affiliations:** ^1^Department of Applied Microbiology and Biotechnology, Microbiome Laboratory, Yeungnam UniversityGyeongsan, South Korea; ^2^Department of Energy and Materials Engineering, Dongguk University, SeoulSeoul, South Korea; ^3^National Science Museum, Ministry of Science, ICT and Future PlanningDaejeon, South Korea; ^4^Department of Forestry, North Eastern Regional Institute of Science and TechnologyNirjuli, India; ^5^Department of Microbiology, Dolphin (PG) College of Science & AgricultureFatehgarh Sahib, India; ^6^College of Pharmacy, Chungnam National UniversityDaejeon, South Korea

**Keywords:** antimicrobial effect, *Rubia philippinensis*, (+)-lariciresinol, foodborne pathogens, scanning electron microscopy

## Abstract

This study was undertaken to assess the antibacterial potential of a polyphenolic compound (+)-lariciresinol isolated from *Rubia philippinensis* against selected foodborne pathogens *Staphylococcus aureus* KCTC1621 and *Escherichia coli* O157:H7. (+)-Lariciresinol at the tested concentrations (250 μg/disk) evoked a significant antibacterial effect as a diameter of inhibition zones (12.1–14.9 mm) with minimum inhibitory concentration (MIC), and minimum bactericidal concentration values of 125–250 and 125–250 μg/mL, respectively. Furthermore, (+)-lariciresinol at MIC showed reduction in bacterial cell viabilities, efflux of potassium (K^+^) ions and release of 260 nm materials against *E. coli* O157:H7 and *S. aureus* KCTC1621. Moreover, deteriorated cell wall morphology of *E. coli* O157:H7 and *S. aureus* KCTC1621 cells treated with (+)-lariciresinol at MIC further confirmed its inhibitory effect against the tested pathogens, suggesting it to be an alternative means of antimicrobials.

## Introduction

Food industry and regulatory authorities are more concerned about the safety of food, and its contamination by foodborne pathogenic bacteria (Al-zorekya and Al–Taher, 2015). Consumer awareness on the knowledge of quality food information has raised greater concern to use high quality food products due to possible contamination by foodborne pathogenic bacteria. Among foodborne pathogens, *Staphylococcus aureus* and *Escherichia coli* O157:H7 have posed serious concerns to consumers due to their reported foodborne outbreaks and foodborne illnesses (Awaisheh et al., 2013). In the USA, about 75 and 68% cases account for foodborne outbreaks and reported cases of foodborne illnesses, respectively (Scot, 2003). Meat associated foodborne illnesses in Canada costs about five hundred million dollars for their cure (Oussalah et al., 2007). Furthermore, failure to control foodborne pathogens using current preservation techniques has raised a serious concern to explore new and natural classes of antimicrobials to combat against serious foodborne pathogens (Awaisheh and Ibrahim, 2009). Recently, it has been found that currently available preservation techniques have not provided optimal results to control or retard the growth of foodborne and food spoilage pathogens *in vivo* (Aneja et al., 2014).

On the other hand, current scenario in drug discovery and development has consistently showed development of resistance among the foodborne pathogens to the commercially available antibiotics (Militello et al., 2011). Though some synthetic antimicrobials have shown profound efficacy to control foodborne pathogens (Shakiba et al., 2011; Shen et al., 2014), they have shown to exhibit several side effects as a serious human-health threatening, including toxic, carcinogenic, and teratogenic effects (Hsouna et al., 2011). This has caused an alarming threat to the consumers to acquire contamination free food or food products.

For decades, plants or plant-based phyto-constituents have been used as alternative means for the prevention and treatment of various diseases, both in modern and traditional medicine system (Pandey and Rizvi, 2009; Vauzour et al., 2010; Bajpai et al., 2015). Plant extracts from *Trachyspermum ammi* (L.) were found to be effectivive against different foodborne pathogens (Paul et al., 2011). Also, olive and oregano derived extracts found to effective against different foodborne pathogens includings, *S. aureus, S. enterica, L. monocytogenes*, and *E. coli* O157:H7 (Friedman et al., 2013). Polyphenols and bioactive compounds extracted from *Camellia sinensis* showed significant inhibitory effects agaist *E. coli* (Noormandi and Dabaghzadeh, 2015). In the present study, a polyphenolic lignan, (+)-lariciresinol isolated from the roots of *Rubia philippinensis* was assessed for its antimicrobial efficacy against selected foodborne pathogens, *Staphylococcus aureus* and *Escherichia coli* O157:H7.

## Materials and Methods

### Chemicals and Reagents

The nutrient broth (NB) medium was purchased from Difco (USA). Other chemicals and reagents used were of very pure and high analytical grade. Test samples of (+)-lariciresinol isolated from *R. philippinensis* were prepared in 1% dimethyl sulfoxide (DMSO) (Sigma-Aldrich, Germany). Spectrophotometric measurements were made using an enzyme-linked immunosorbent assay (ELISA) instrument (Tecan, Infinite M200, Männedorf, Switzerland) was used.

### Test Foodborne Pathogens

Two selected foodborne pathogenic bacteria one Gram-positive (*Staphylococcus aureus* KCTC1621), and one Gram-negative (*Escherichia coli* O157:H7, shiga-toxin producing bacterium) were procured from the Korean Collection for Type Cultures (KCTC, Korea) and used in this study. All the bacterial strains were grown in the NB and incubated at 37°C. The bacterial strains were maintained on nutrient agar slants at 4°C.

### Plant Materials

The roots of *Rubia philippinensis* were collected from Bidoup-Nui Ba National Park, Lamdong province, Vietnam in July 2013, and identified by Dr. Phuong Thien Thuong at the Department of Pharmaceutical Analysis and Herbal Standardization, National Institute of Medicinal Materials, Vietnam. A voucher specimen was deposited at the herbarium of the National Institute of Medicinal Materials, Hanoi, Vietnam, as well as the Laboratory of Pharmacognosy at the College of Pharmacy, Chungnam National University, Daejeon, Korea.

### Extraction, Isolation, and Characterization of (+)-Lariciresinol

(+)-Lariciresinol was obtained from the roots of *R. philippinensis* by chromatographic methods. Briefly, the ethanol extract of *R. philippinensis* (150 g) was suspended in H_2_O (1.5 L) and partitioned with CH_2_Cl_2_ (2 L × 3) to yield CH_2_Cl_2_ extract. The CH_2_Cl_2_-soluble fraction (50 g) was subjected to silica gel VLC and eluted with *n*-hexane-EtOAc (20:1, 10:1, 5:1, 3:1, 2:1) and CHCl_3_-MeOH (8:1) to afford six fractions (D-1 → D-6). Fraction D-6 (10 g) was divided into 11 sub-fractions (D-6-1 → D-6-11) using MPLC with a gradient of MeOH-H_2_O (10:90 → 100:0, 7 L). (+)-Lariciresinol (*t*_R_ 33.0 min, 31 mg) was obtained from D-6-3 (360 mg) by HPLC model 1200 using a Zorbax SB-C18 analytical column (4.6 mm × 150 mm, 5 μm particle size), Agilent, Germany eluting with MeOH-H_2_O (45:55, 4 mL/min, UV 254 nm). (+)-Lariciresinol: brownish amorphous powder, ^1^H NMR (300 MHz, methanol-*d*_4_): 2.38 (1H, m, H-8), 2.47 (1H, dd, *J* = 13.1, 11.7, H_a_-7’), 2.72 (1H, m, H-8’), 2.91 (1H, dd, *J* = 13.1, 4.5, H_b_-7’), 3.63 (1H, dd, *J* = 10.8, 6.6, H_a_-9), 3.72 (1H, dd, *J* = 8.1, 6.1, H_a_-9’), 3.82 (1H, overlapped, H_b_-9), 3.97 (1H, dd, *J* = 8.1, 6.1, H_b_-9’), 4.75 (1H, d, *J* = 6.9, H-7), 6.63 (1H, dd, *J* = 8.0, 1.2, H-6’), 6.73 (1H, d, *J* = 8.0, H-5’), 6.78 (3H, overlapped, H-6, H-5, H-2’), 6.91 (1H, d, *J* = 1.2, H-2); ^13^C NMR (75 MHz, methanol-*d*_4_): 135.7 (C-1), 110.6 (C-2), 148.9 (C-3), 146.9 (C-4), 116.0 (C-5), 119.8 (C-6), 83.9 (C-7), 53.9 (C-8), 60.4 (C-9), 133.5 (C-1’), 113.4 (C-2’), 148.9 (C-3’), 145.7 (C-4’), 116.2 (C-5’), 122.1 (C-6’), 33.6 (C-7’), 43.8 (C-8’), 73.4 (C-9’), 56.3, 56.3 (2 × OCH_3_-3,3’).

### Determination of Antibacterial Activity of (+)-Lariciresinol

Standard agar diffusion method was used for antibacterial assay (Bajpai and Kang, 2010). Petri plates were prepared by pouring 20 ml of LB medium and allowed to solidify. Plates were dried, and 100 μL of standardized inoculum containing approximately 10^7^ CFU/mL of bacterial suspension was poured and uniformly spread. The inoculum was allowed to dry for 5 min. The compound was dissolved in 5% DMSO, and a Whatman No. 1 sterile filter paper disk (6 mm diameter) was impregnated with 50 μL of the compound (+)-lariciresinol corresponding to 250 μg/disk. Negative controls were prepared using the same solvent that was employed to dissolve the sample. The plates were incubated at 37°C for 24 h in a bacterial incubator chamber (Thermo Scientific Ltd., Korea). Antibacterial activity was evaluated by measuring the diameters of the zones of inhibition against the tested bacteria. Each assay in this experiment was replicated three times.

### Determination of Minimum Inhibitory Concentration (MIC) and Minimum Bactericidal Concentratin (MBC) of (+)-Lariciresinol

The MIC of (+)-lariciresinol was tested by the two-fold serial dilution method (Bajpai et al., 2013). The (+)-lariciresinol was first dissolved in 5% DMSO, and incorporated into NB medium for bacterial pathogens to obtain a concentration of 500 μg/mL, and serially diluted in NB broth medium to achieve 250, 125, 62.5, 31.25, 15.62, and 7.81 μg/mL, respectively. A 10 μL standardized suspension (approximately 10^7^ CFU/mL) of each tested organism was transferred to each tube. The control tubes containing only bacterial suspensions were incubated at 37°C for 24 h in a bacterial incubator chamber (Thermo Scientific Ltd., Korea). The lowest concentration of (+)-lariciresinol, which did not show any visible growth of test organisms after macroscopic evaluation, was determined as MIC, which was expressed in μg/mL. Further, the concentrations showing complete inhibition of visual growth of bacterial pathogens were identified, and 50 μL of each culture broth was transferred onto the agar plates and incubated for specified time and temperature as mentioned above. The complete absence of growth of bacterial colony forming unit (CFS) on the agar surface is the lowest concentration of the sample and was defined as MBC. Each assay in this experiment was replicated three times.

### Determination of the Effect of (+)-Lariciresinol on Bacterial Viabilities

Freshly grown bacterial colonies of the selected pathogenic bacteria were inoculated in NB medium at 37°C for 24 h, and then bacterial cultures were serially diluted to 10^7^ CFU/mL (Shin et al., 2007). To determine the effect of (+)-lariciresinol on cell viabilities, each of the tubes containing the bacterial suspension (10 μL; approximately 10^7^ CFU/mL) of *S. aureus* KCTC1621 and *E. coli* O157:H7 was inoculated with 100 μL of (+)-lariciresinol at its MIC in 890 μL NB broth at 37°C. Samples for viable cell counts were taken out at 0, 40, 80, 120, 160, and 200 min time intervals. Viable plate counts were monitored on NB agar as we previously described (Bajpai et al., 2013). Colonies were counted after incubation for 24 h at 37°C in a bacterial incubator chamber (Thermo Scientific Ltd., Korea). The controls were inoculated without (+)-lariciresinol for each pathogen using the same experimental condition. Assay were performed in triplicate.

### Determination of the Effect of (+)-Lariciresinol on Potassium (K^+^) Ion Efflux

Effect of lariciresinol on the efflux of potassium ion from the tested pathogens was determined according to our previously reported method (Bajpai et al., 2013). The concentrations of free potassium ions from *S. aureus* KCTC1621 and *E. coli* O157:H7 cell suspensions were measured by a photometric procedure after the exposure of cells to at MIC in sterile peptone water using the Calcium/Potassium ions kit (Quantofix, GmbH, Wiesbaden, Germany). Similarly control was also tested without adding CFS. Results were expressed as the release of extracellular free K^+^ ion (mmol/L) in the growth media at pre-established incubation intervals for 0, 30, 60, 90, and 120 min.

### Determination of the Effect of (+)-Lariciresinol on the Release of 260-nm Absorbing Cellular Materials

The measurement of the release of 260-nm-absorbing materials from *S. aureus* KCTC1621 and *E. coli* O157:H7 cells was carried out in aliquots of 2 mL of the bacterial inocula in sterile peptone water (0.1 g/100 mL). The reaction solution was added of MIC of (+)-lariciresinol and incubated at 37°C. At 0, 30, and 60 min time interval of treatment, cells were centrifuged at 3,500 × g, and the absorbance of the obtained supernatant was measured at 260 nm using a 96-well plate ELISA reader (Magellan5, TECAN, Korea) (Bajpai et al., 2013). Similarly control was also tested without adding (+)-lariciresinol. Results were expressed in terms of optical density (OD) of 260-nm absorbing materials in each interval with respect to the ultimate time.

### Determination of the Effect of (+)-Lariciresinol on the Cell Wall Morphology of Foodborne Pathogens

Scanning electron microscopic (SEM) study was executed according to Kim et al. (2007) to examine the effects of (+)-lariciresinol on the morphological changes in the cell wall of the selected pathogens, *S. aureus* KCTC1621 and *E. coli* O157:H7 at MIC. Control samples were prepared without (+)-lariciresinol. Microscopic examination was performed using a S-4300 SEM Analyzer (Hitachi, Japan).

### Statistical Analysis

The data were statically analyzed using software package SPSS v.19.0 statistical software package (SPSS Inc., Chicago, IL, USA). All experiments were performed in triplicate and results were expressed the mean ± SD following one-way ANOVA statistical analysis coupled with Duncan’s multiple test.

## Results

### Identification and Characterization of (+)-Lariciresinol

**FIGURE 1:**
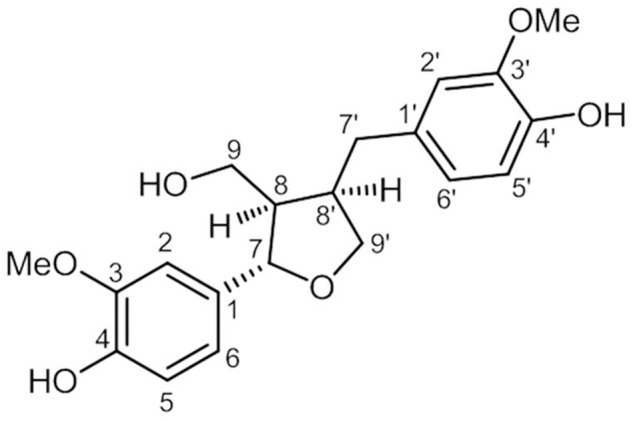
**The chemical structure of (+)-lariciresinol isolated from *Rubia philippinensis***.

The ^1^H NMR data of purified compound, [α]_D_ +17 (*c* 0.05, MeOH), displayed two pairs of aromatic ring systems at δ_H_ 6.63 (1H, dd, *J* = 8.0, 1.2, H-6’), 6.73 (1H, d, *J* = 8.0, H-5’), 6.78 (3H, overlapped, H-6, H-5, H-2’), and 6.91 (1H, d, *J* = 1.2, H-2). In addition, an oxymethylene moiety at δ_H_ 3.72 (1H, dd, *J* = 8.1, 6.1, Ha-9’), 3.97 (1H, dd, *J* = 8.1, 6.1, Hb-9’), two methine groups at δ_H_ 2.38 (1H, m, H-8) and 2.72 (1H, m, H-8’), and an oxymethine at δ_H_ 4.75 (1H, d, *J* = 6.9, H-7) indicating a tetrahydrofuran substructure were observed in the ^1^H NMR data as well. The ^13^C NMR spectrum showed 18 signals for a typical lignan derivative (**Figure 1**).

### Antibacterial Activity

In this assay, presence or absence of inhibition zones determined the antibacterial potential of (+)-lariciresinol against the test foodborne pathogenic bacteria. As presented in **Table 1**, (+)-lariciresinol exhibited a considerable amount of inhibitory effect against the tested foodborne pathogens. It was observed that (+)-lariciresinol exerted a consistent antibacterial effect as diameters of inhibition zones against both Gram-positive and Gram-negative bacteria, which were found in the range of 12.1–14.9 mm (**Table 1**). The NB broth, used as a negative control, had no inhibitory effect on the growth of the tested pathogens.

**Table 1:** Antibacterial activity of (+)-lariciresinol against foodborne pathogens *S. aureus* KCTC1621 and *E. coli* O157:H7.

Pathogens	(+)-Lariciresinol		
	**Zones of inhibition^a^**	**Susceptibility**	
		**MIC^b^**	**MBC^c^**
*Staphylococcus aureus* KCTC1621	14.9 ± 0.2	125	125
*Escherichia coli* O157:H7	12.1 ± 0.3	250	250

*^a^Diameters of inhibition zones in millimeter; ^b^Minimum inhibitory concentration (values in μg/mL); ^c^Minimum bactericidal concentration (values in μg/mL). All values were expressed as mean ± SD of three parallel measurements (*n* = 3). Values in the same column with different superscripts are significantly different according to Duncan’s Multiple Range Test (*P* < 0.05). Zones of inhibition around the disk were measured in millimeter (mm) using a Vernier’s caliper.*

### MIC and MBC

In this assay, test foodborne pathogens displayed different susceptibility rates to (+)-lariciresinol, and (+)-lariciresinol exhibited potent inhibitory effect as confirmed by its different MIC and MBC values against both the tested pathogens. As a result, the MIC and MBC values of (+)-lariciresinol against the tested foodborne pathogens were found in the range of 125–250 and 125–250 μg/mL, respectively (**Table 1**). In this assay, (+)-lariciresinol exhibited consistent antibacterial effect, however, the inhibitory effect of (+)-lariciresinol was more profound against Gram-positive bacterium than Gram-negative bacterium.

### Effect on Bacterial Cell Viability

**FIGURE 2:**
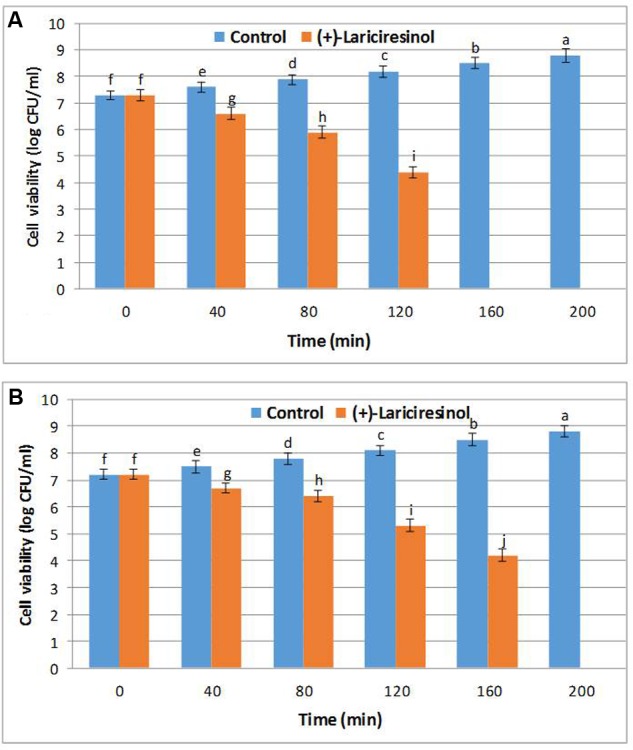
**Effect of (+)-lariciresinol on the viability of the tested pathogenic bacteria of *S. aureus* KCTC1621 (A)** and *E. coli* O157:H7 **(B)**. Control without treatment. Data are expressed as mean ± SD (*n* = 3). Values in the same column with different superscripts are significantly different according to Duncan’s Multiple Range Test (*P* < 0.05).

In this assay, (+)-lariciresinol when inoculated at MIC, exhibited significant inhibitory effects against the growth of tested pathogens, *S. aureus* KCTC1621 and *E. coli* O157:H7, as confirmed by reducing pattern in the bacterial cell viabilities (**Figure 2**). Exposure to (+)-lariciresinol for 0 to 80 min did not elicit severe inhibition of cell viability, but remarkable declines in the cell viable counts of *S. aureus* KCTC1621 and *E. coli* O157:H7 was observed after exposure to (+)-lariciresinol for 160 min. Interestingly, the exposure to (+)-lariciresinol for 200 min completely inhibited the cell viabilities of both tested pathogens (**Figure 2**).

### K+ Ion Efflux

**FIGURE 3:**
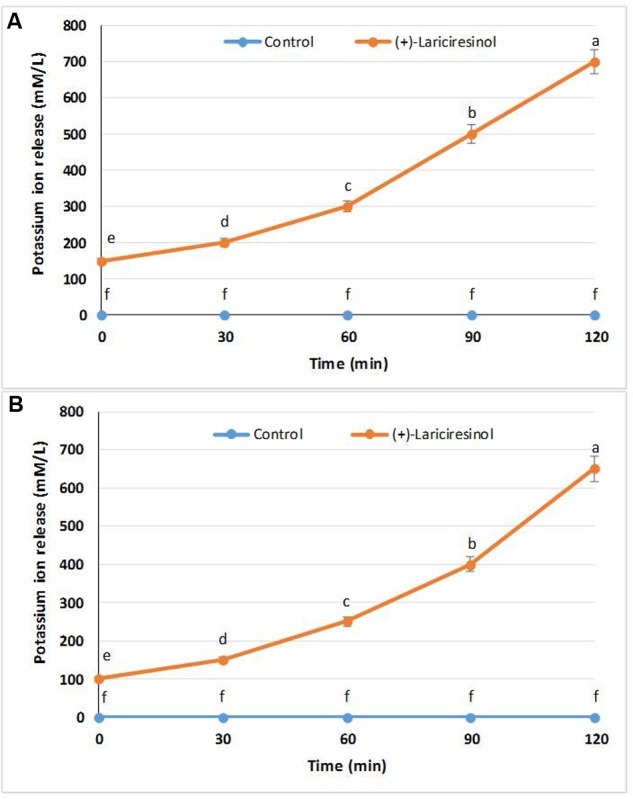
**Effect of (+)-lariciresinol on the release rate of extracellular K^+^ ions from *S. aureus* KCTC1621 (A)** and *E. coli* O157:H7 **(B)**. Data are expressed as mean ± SD (*n* = 3). Values in the same column with different superscripts are significantly different according to Duncan’s Multiple Range Test (*P* < 0.05).

Release of extracellular K+ ions from the bacterial cells when treated with a suitable antimicrobial could be an indication of the establishment of the bactericidal effect of the tested compound. Interestingly, in this assay, (+)-lariciresinol was able to confirm its bactericidal effect against both the tested pathogens, *S. aureus* KCTC1621 and *E. coli* O157:H7 as confirmed by the marked release of free K^+^ ions from the treated bacterial cells as compared to non-treated cells used as a control (**Figure 3**). The (+)-lariciresinol (at MIC) allowed the K+ ion release of (150–700 mmol/L) and (100–650 mmol/L) against *S. aureus* KCTC1621 and *E. coli* O157:H7, respectively (**Figures 3A,B**).

### Release of 260 nm Absorbing Cellular Materials

**FIGURE 4:**
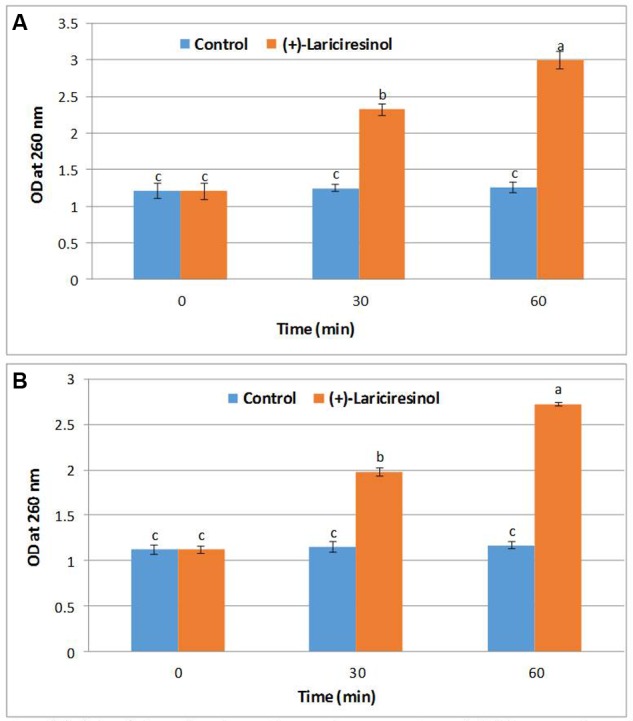
**Effect of (+)-lariciresinol on the release rate of 260-nm absorbing material from *S. aureus* KCTC1621 (A)** and *E. coli* O157:H7 **(B)**. Data are expressed as mean ± SD (*n* = 3). Values in the same column with different superscripts are significantly different according to Duncan’s Multiple Range Test (*P* < 0.05).

In this assay, we determined the release of 260-nm absorbing materials from the bacterial cells when treated with (+)-lariciresinol at MIC. Since the excessive release of 260 nm materials (DNA and RNA) may lead to irreversible injury to bacterial cells, and (+)-lariciresinol caused a significant effect on the bacterial cells (**Figure 4**). Bacterial cells when treated with (+)-lariciresinol allowed significantly higher release of 260 nm materials from *S. aureus* KCTC1621 (**Figure 4A**) and *E. coli* O157:H7 (**Figure 4B**) when compared with control cells as confirmed by a significant difference in optical densities observed between treatment (2.72–2.99) and control (1.12–1.26) cells measured at 260 nm. In contrast, no changes in the OD of control cells of tested pathogens were observed during the study.

### Observation of Morphological Changes in Bacterial Cell Wall

**FIGURE 5:**
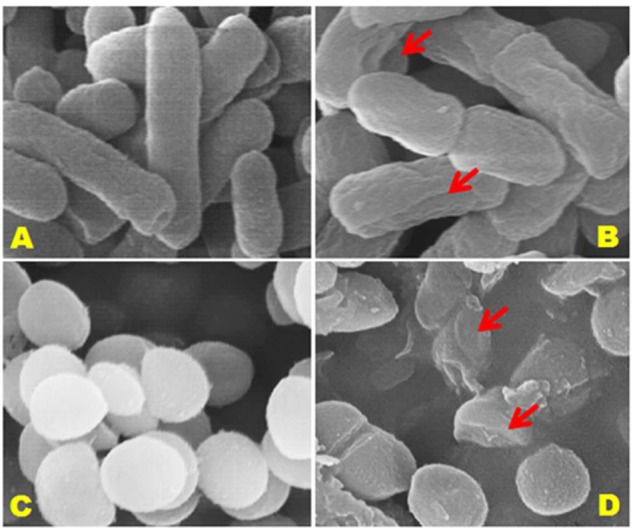
**Scanning electron microscopic (SEM) analysis of *E. coli* 0157:H7 and *S. aureus* KCTC1621 cells treated with (+)-lariciresinol at MIC.** Controls **(A,C)** showing a regular and smooth surface; whereas treated cells **(B,D)** arrows showing disruption and cell lysis, respectively.

Since exposure to an antimicrobial agent may lead to disruption of bacterial cell wall, we turned to SEM analysis to investigate further the effect of (+)-lariciresinol on the cell wall physiologies and morphologies of *S. aureus* KCTC1621 and *E. coli* O157:H7 cells (**Figure 5**). As was expected, control bacterial cells not exposed the result of (+)-lariciresinol had regular smooth surfaces (**Figures 5A,C**), whereas those treated with (+)-lariciresinol at MIC showed cell wall damage and lysis (**Figures 5B,D**).

## Discussion

The NMR spectrum of the tested compound showed 18 signal derivatives for a typical lignin. On the basis of NMR spectroscopic data analyses and specific rotation, the compound was identified as (+)-lariciresinol (Xie et al., 2003).

The current finding showed the consistent inhibitory effect of (+)-lariciresinol as diameters of inhibition zones for both the tested pathogens. Similarly, two polyphenols proauthocyanidin (I) and biflavanoid (III) isolated from the stem bark of *Garcinia indica* were found to display antibacterial effects against foodborne pathogenic bacteria, including *S. aureus, Salmonella typhi*, and *E. coli* (Lakshmi et al., 2011). In addition, a number of plants rich in polyphenolic compounds have been found to exhibit remarkable antimicrobial effects against a number of pathogenic microbes including foodborne pathogens (Saravanan and Parimelazhagan, 2014).

Moreover, some of the pure compounds exhibit greater inhibitory effect against a diverse range of pathogenic bacteria. In this study, the Gram-positive bacterium was found to be more susceptible to the (+)-lariciresinol than Gram-negative bacterium. The hydrophilic cell wall structure of Gram-negative bacterium is constituted essentially of lipopolysaccharide that blocks the penetration of hydrophobic components and avoids the accumulation of (+)-lariciresinol in the target cell membrane (Bezic et al., 2003; Bajpai et al., 2013). The single membrane of Gram-positive bacteria is considerably more accessible to permeation by (+)-lariciresinol in the target sites, and similar results were also reported by other researchers (Patra and Baek, 2016). This might be the reason that Gram-positive bacterium was found to be more sensitive to the (+)-lariciresinol than Gram-negative bacterium.

The previous studies, on bioactive compounds from *M. glyptostroboides* (Bajpai and Kang, 2010), polyphenols from medicinal plants *Burkina Faso, Combretum micranthum, Khaya senegalensis, Pterocarpus erinaceus*, and *Sida acuta*, (Karou et al., 2005) and phytochemical compounds of olive and oregano (Friedman et al., 2013) have shown enormous inhibitory potential against foodborne pathogens with a considerable amount of MIC and MBC values, which supports the current findings.

This study shows that exposure to (+)-lariciresinol was able to reduce viable cell counts of both *S. aureus* KCTC1621 and *E. coli* O157:H7. Previous reports have confirmed the inhibitory effects of various plant-based phytochemicals against foodborne pathogenic bacteria (Bajpai and Kang, 2010). Also, plants or their oils rich in polyphenolic compounds have shown significant results of antimicrobial efficacy through the reduction in cell viabilities of various tested foodborne pathogens (Patra and Baek, 2016).

The release of extracellular K+ ions from bacterial cells is an indicator of bactericidal effect of compound used for the treatment. Other antimicrobials compounds tested for their efficacy on K+ ion release have also shown similar findings when tested against specific foodborne pathogens (Patra et al., 2015). Bacterial plasma membrane acts as a barrier of permeabilization to the release of necessary electrolytes, including potassium ions. Since impermeability to small ions is regulated by the membrane chemical nature and structural composition, increasing release of K^+^ ions from the bacterial cells clearly demonstrates disruption of the plasma membrane, thereby confirming the antimicrobial role of (+)-lariciresinol. Similar findings were observed previously by Cox et al. (2001).

The results of this study confirmed that increased release of 260 nm material from the bacterial cells when treated with a specific antibacterial agent (+)-lariciresinol caused a deleterious effect indicating structural damage to plasma membrane thus causing cell death, as also confirmed previously (Patra and Baek, 2016). These findings reinforce the suggestions that monitoring of release of nucleic acid from *S. aureus* KCTC1621 and *E. coli* O157:H7 might be a more sensitive indicator of membrane damage and loss of membrane integrity. Similarly, Souza et al. (2013) reported the effect of polyphenolic compounds carvacrol and thymol on the release of nucleic acid and other cell electrolytes and confirmed their inhibitory effects on membrane permeabilization against a number of strains of a foodborne pathogen *S. aureus*.

In this study, SEM analysis showed marked morphological changes to the cell walls of *S. aureus* KCTC1621 and *E. coli* O157:H7 resulting in cell wall deformation by (+)-lariciresinol. Consistent with our findings, Alwash et al. (2013) also reported the effect of polyphenolic flavonoid Kaempferol-3-*O*-(2′,6′-di-*O*-*trans*-*p*-coumaroyl)-beta-D-glucopyranoside inducing such morphological alterations in several pathogenic microbes including foodborne pathogens. These morphological alterations may be due to aberrations in membrane lipid composition, altered membrane fluidity, and/or membrane integrity resulting in cell wall lysis and loss of intracellular dense material (Cui et al., 2015).

## Conclusion

This study reports characterization of a phenylpropanoid lignan, (+)-lariciresinol isolated from the roots of *Rubia philippinensis* which exhibited significant inhibitory effects against selected foodborne pathogenic bacteria in different *in vitro* assays. More specifically, this study confirms that (+)-lariciresinol exerted its inhibitory effect through permeabilization of the cell membrane associated with generalized membrane-disrupting effects. These findings reinforce the conclusion that (+)-lariciresinol exhibiting a significant antibacterial activity, can be used as a natural antimicrobial agent for using in the food industry to control or retard the growth of foodborne pathogens.

## Author Contributions

VB design and conceive the experiment and prepare the manuscript. SS assist during the experiments design and manuscript preparation. WP and PaK help in manuscript preparation. JL, PrK, and MN did the editing and finalize the manuscript for submission.

## Conflict of Interest Statement

The authors declare that the research was conducted in the absence of any commercial or financial relationships that could be construed as a potential conflict of interest.
